# Does Head Tilt Influence Facial Appearance More Than Head Turn?

**DOI:** 10.18502/jovr.v18i3.13778

**Published:** 2023-07-28

**Authors:** Mohamad Reza Akbari, Masoud Khorrami-Nejad, Haleh Kangari, Mohsen Heirani, Alireza Akbarzadeh Baghban, Kiana Raeesdana, Babak Masoomian

**Affiliations:** ^1^Translational Ophthalmology Research Center, Farabi Eye Hospital, Tehran University of Medical Sciences, Tehran, Iran; ^2^School of Rehabilitation, Tehran University of Medical Sciences, Tehran, Iran; ^3^School of Rehabilitation, Shahid Beheshti University of Medical Sciences, Tehran, Iran; ^4^Proteomics Research Center, Department of Biostatistics, School of Allied Medical Sciences, Shahid Beheshti University of Medical Sciences, Tehran, Iran; ^5^School of Optometry, University of Montreal, Montréal, Canada

**Keywords:** Duane Retraction Syndrome, Facial Asymmetry, Head Tilt, Head Turn, Upshoot In Adduction

## Abstract

**Purpose:**

To evaluate the frequency of facial asymmetry parameters in patients with head tilt versus those with head turn.

**Methods:**

This cross-sectional comparative study was performed on 155 cases, including 58 patients with congenital pure head turn due to Duane retraction syndrome (DRS), 33 patients with congenital pure head tilt due to upshoot in adduction or DRS, and 64 orthotropic subjects as the control group. The facial appearance was evaluated by computerized analysis of digital photographs of patients' faces. Relative facial size (the ratio of the distance between the external canthus and the corner of the lips of both face sides) and facial angle (the angular difference between a line that connects two external canthi and another line that connects the two corners of the lips) measured as quantitative facial parameters. Qualitative parameters were evaluated by the presence of one-sided face, cheek, and nostril compression; and columella deviation

**Results:**

The facial asymmetry frequency in patients with head tilt, head turn, and orthotropic subjects was observed in 32 (97%), 50 (86.2%), and 22 (34.3%), respectively (*P* < 0.001). In patients with head tilt and head turn, the mean facial angle was 1.78º ± 1.01º and 1.19º ± 0.84º, respectively (*P *= 0.004) and the mean relative facial size was 1.027 ± 0.018 and 1.018 ± 0.014, respectively (*P *= 0.018). The frequencies of one-sided nostril compression, cheek compression, face compression, and columella deviation in patients with pure head tilt were found in 19 (58%), 21 (64%), 19 (58%), and 19 (58%) patients, respectively, and in patients with pure head turn the frequencies were observed in 42 (72%), 37 (63%), 27 (47%), and 43 (74%), respectively. All quantitative and qualitative facial asymmetry parameters and facial asymmetry frequencies were significantly higher in head tilt and head turn patients as compared to the control group (*P* < 0.001).

**Conclusion:**

All facial asymmetry parameters in patients with head tilt and head turn were significantly higher than orthotropic subjects. The quantitative parameters such as relative facial size and facial angle were significantly higher in patients with pure head tilt than pure head turn. The results revealed that pure head tilt was associated with a higher prevalence of facial asymmetry than pure head turn.

##  INTRODUCTION

Abnormal head posture is defined as a condition in which the head is deviated out of the normal primary straight position and angled relative to the body either in the fronto-posterior (head tilt), vertical (head turn), or horizontal (chin elevation or depression) axes.^[1,2]^ Abnormal head posture for compensation of binocular vision deficiencies from nystagmus, superior oblique palsy, Duane retraction syndrome (DRS), and other ocular diseases is referred to as ocular torticollis.^[3,4]^

Superior oblique palsy is one of the main causes of ocular abnormal head posture and the most common type of extraocular muscle palsies.^[3,4][5][6]^Previous studies reported that in patients with superior oblique palsy, abnormal head posture is often observed as pure head tilt to the contralateral side of the palsy.^[3,4]^

Another common cause of abnormal head posture is DRS.^[3,4][5]^In these patients, head turn as a compensatory mechanism assists the patients in preventing diplopia and obtaining binocular single vision. Esotropic DRS patients (type I), due to the limitation of abduction, turn their heads toward the same side of the affected eye, however, exotropic DRS patients have limitations of adduction and the head turn is toward the opposite side of the affected eye.^[7,8]^

Ocular torticollis has been associated with the development of facial asymmetry. Most patients with congenital causes of ocular torticollis also have facial asymmetry.^[7,8][9][10][11][12]^ The main reason for applying early treatment to ocular abnormal head posture is to try to prevent subsequent muscular changes and facial asymmetry.^[9]^

Longstanding abnormal head posture can affect the symmetry of the neck,^[13]^ face, cheek, nostril, nasal tip, and columella.^[7,8][10]^ The presence of facial asymmetry in an adult can help to confirm the congenital source of abnormal head posture and it also avoids unnecessary neurologic evaluations. In patients with ocular torticollis, resolving abnormal head posture by strabismus surgery may help to prevent facial asymmetry.^[7,9]^

The presence and development of facial asymmetry depends on the type and duration of the abnormal head posture.^[7,11]^ Based on two previous studies, the high frequency of abnormal quantitative and qualitative facial asymmetry parameters was observed in patients with ocular abnormal head posture in comparison with orthotropic subjects.^[8,10]^ However, to the best of our knowledge, the comparison of facial asymmetry parameters frequency in patients with head tilt versus those with head turn has not been evaluated yet. The main purpose of this study was to compare the frequency of quantitative and qualitative facial asymmetry parameters in patients with head tilt versus head turn and compare with orthotropic subjects.

##  METHODS

This cross-sectional comparative case series study was performed on 155 subjects (91 patients and 64 orthotropic subjects) at Farabi Eye Hospital, Iran, from October 2019 to September 2020. Ninety-one patients with ocular torticollis were divided into two groups: 58 patients with pure head turn and 33 with pure head tilt. In addition, 64 orthotropic subjects were selected as a control group from the siblings or cousins of the patients. All three groups were age-matched (*P *= 0.402).

The Institutional Review Board of Shahid Beheshti University of Medical Sciences approved the study protocol (Ethics code: IR.SBMU.RETECH.REC.1399.228). It was performed according to the tenets of the Declaration of Helsinki. The study's aim and method were clearly described, and then written informed consent to publish patients' findings and face pictures were obtained from all participants or their parents if they were under the age of 18.

The inclusion criteria for the “head tilt” and “head turn” groups were constant pure head tilt or pure head turn due to unilateral upshoot in adduction or DRS. The inclusion criteria for the control group were subjects being siblings or cousins of patients who did not have any types of abnormal head posture and strabismus. The exclusion criteria for both the patients and control groups were the existence of any type of skeletal and muscular facial abnormalities such as craniofacial anomalies, plagiocephaly syndromes, and any motor and mental disabilities. Patients with a history of severe head trauma and any facial cosmetic surgery or nonsurgical facial rejuvenation were also excluded from this study. As previous studies reported a distance-near disparity in abnormal head posture due to nystagmus,^[14]^ patients with this cause of abnormal head posture were also excluded from the study. Vision in all cooperative participants was better than 0.2 LogMAR in both eyes.

Routine eye examinations such as measurements of refractive errors and vision were assessed. Dilated fundoscopic examinations were performed for all patients and orthotropic individuals. The angle of ocular deviation was recorded with alternate prism cover tests at far and near distances. The exact cause of abnormal head posture was determined by using version and duction tests. A diagnosis of DRS was made based on observing adduction or abduction limitations, overshoot, and globe retractions with the narrowing of the palpebral fissure.^[15]^ Upshoot in adduction was diagnosed based on the result of the Parks-Bielschowsky three-step test (hypertropia in the central gaze that increased in ipsilateral head tilt and on the contralateral gaze). A congenital etiology for upshoot in adduction was confirmed based on the patient's childhood photographs, absence of diplopia, and long-term history of strabismus.^[16]^

The exact manifestation of abnormal head posture was determined by direct observation from different axes. “Head tilt” was defined as rotating the head around the anterioposterior axis of the skull and “head turn” as the rotation of the anterioposterior axis of the skull from the normal position.^[1,2]^

In this study, facial asymmetry assessment was based on the two following methods.

### Quantitative Method

In this method, a picture was taken of the face when the head was completely straight. The picture was analyzed by CorelDRAW Graphics Suite X7 software (Corel Corporation, Ontario, Canada), and two of the following parameters were calculated.

**Figure 1:**
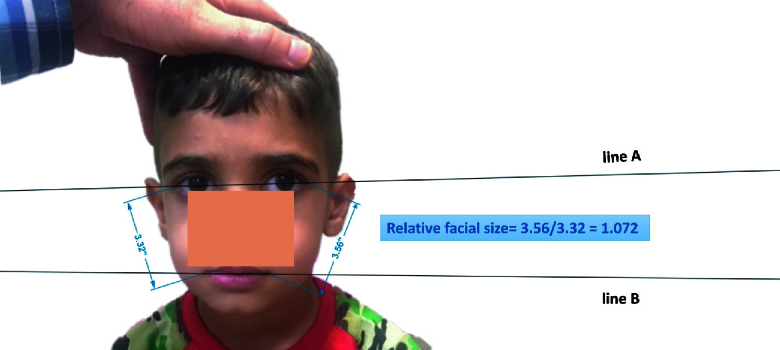
Measurement of relative facial size and facial angle.

Relative facial size: As shown in Figure 1, the ratio of the distance between the external canthus and the corner of the lips of both face sides, named reference lines, was defined as relative facial size. If the distance between the external canthus and the corner of the lips of both face sides was the same, the face was symmetric based on this parameter.^[7,8][12]^ However, when these lines were different, for better comparison of patients, the amount of the longer reference line was divided into the other line and the result was named relative facial size parameter.

Facial angle: As shown in Figure 1, this parameter was measured by calculating the angle between the two following lines; the first line connected two external canthi (line A in Figure 1) and the second line connected the two lips corners (line B in Figure 1). If two lines were parallel, the face was symmetric based on this parameter, however, the presence of an angular difference means facial asymmetry.^[7,8][12]^

All quantitative parameters were calculated three times, and the average was reported as a result.

### Qualitative Method

**Figure 2:**
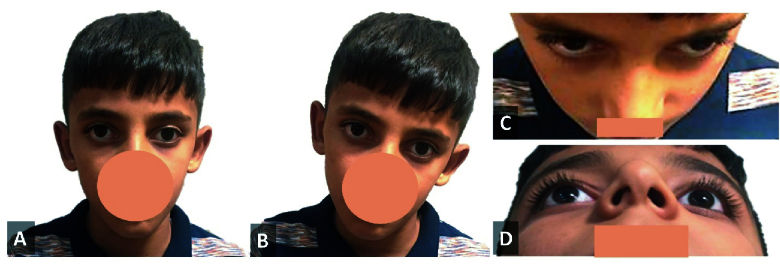
Patient with right upshoot in adduction. (A) Normal head position indicating right hypertropia. (B) Habitual head posture, showing left head tilt. (C) Photo taken from an upward angle, indicating mild left cheek compression. (D) Photo taken from a downward angle, indicating right nostril compression.

**Figure 3:**
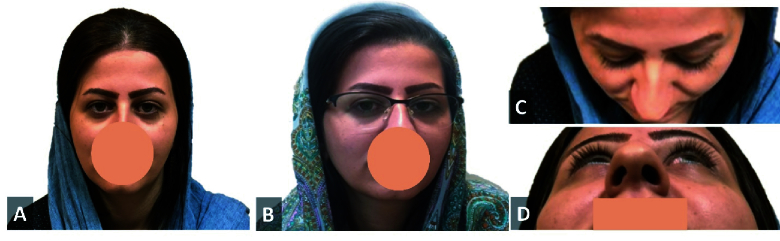
Patient with left esotropic Duane retraction syndrome (type I). (A) Normal head position. (B) Habitual head posture, showing left head turn. (C) Photo taken from above, indicating right cheek compression. (D) Photo taken from downward, indicating right nostril compression.

In this method, facial asymmetry was detected by direct observation of the patients' faces and confirmed with the computerized assessment of patients' photos [Figures 2 & 3]. It included the following facial parameters:

Columella deviation: The presence of columella deviation was recognized by observation of a picture that was taken from a normal primary straight position.^[10]^

Nostril asymmetry: This asymmetry was detected by a picture that was taken from a downward angle and comparison of nostrils sizes [Figures 2D & 3D].^[8]^

Cheek compression: The presence of one-sided cheek compression was recognized by observation in a picture that was taken from an upward angle [Figures 2C & 3C].^[7]^

Face compression: The face side with a shorter reference line was defined as the face compression side.^[7,8]^

All examinations were performed by an expert pediatric ophthalmologist (MR-A) and an expert optometrist (M-KN). Facial asymmetry was defined as the presence of one or more of the following conditions: A relative facial size of > 1.01, facial angle of > +3º or < –3º, and the presence of at least one qualitative asymmetrical parameter on the face (deviation of the columella, compressions of the cheek, face, and nostril on one side of the face in comparison with the other side).^[8,12]^

The collected data were analyzed using SPSS-24 software. Normal data distribution was tested by Shapiro–Wilk, and according to the normal distribution of the data, one-way ANOVA with a Tukey' post hoc analysis test was applied to compare facial angle and relative facial size between patients and control subjects. A graph was drawn using the Microsoft Excel 2019 (Office 365; Microsoft Corporation, Redmond, WA) software and results were presented as the frequency of qualitative facial asymmetry parameters and the presence of facial asymmetry in patients and control groups. Chi squared (χ2) was also used to evaluate the significant differences in facial asymmetry between two sides of the face. Qualitative facial asymmetry parameters, such as columella deviation and unilateral compression of nostril, cheek and face, were considered either as a positive or negative value when they were in the same or opposite direction of the torticollis. The correlation between the affected side of the face or the direction of columella deviation with the side of the torticollis was considered significant when an average value was significantly higher or lower than zero. *P*-values < 0.05 was considered significant.

##  RESULTS

For a better explanation of the results of this study, our findings were classified into three groups and compared among them.

### Group 1: Patients with a pure head tilt

Group 1 consisted of 33 patients with pure head tilt. The mean age of these patients was 14.9 ± 10.3 years (range, 2.5–38 years), which consisted of 8 (24.2%) females and 25 (75.8%) males. All patients had a pure head tilt due to unilateral upshoot in adduction without a head turn or chin down positioning. The side of the head tilt was observed toward the left side in 18 (54.5%) patients and toward the right side in 15 (45.5%) patients (*P *= 0.602).

**Figure 4:**
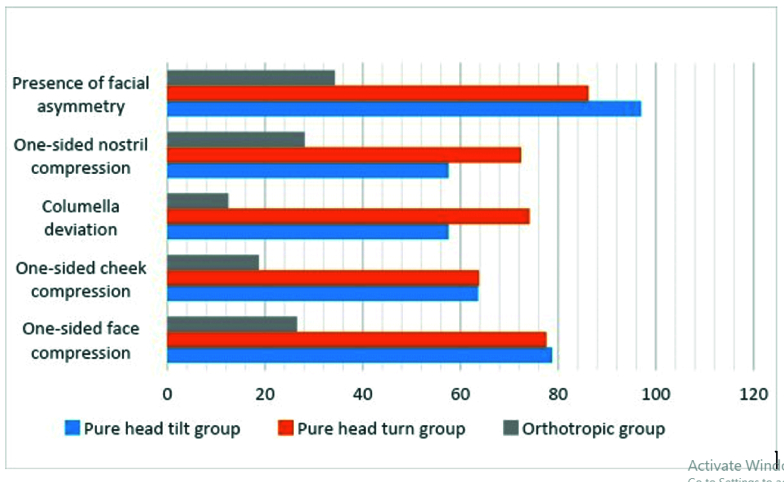
The percentage of qualitative parameters and presence of facial asymmetry in patients with pure head tilt, head turn and control groups.

Facial asymmetry was found in 32 (97%) patients with pure head tilt, and only one (3%) patient had a symmetric face(*P* < 0.001). The mean amounts of quantitative parameters (facial angle and relative facial size) in 33 patients with pure head tilt are shown in Table 1. The frequencies of the presence of facial asymmetry and the qualitative parameters, such as “one-sided nostril compression”, “columella deviation”, “one-sided cheek compression”, and “one-sided face compression” in patients with pure head tilt, are shown in Figure 4.

Thirty-three patients had a pure head tilt – 26 of them had a relative facial size of > 1.01, face compression was on the same side of head tilt in 19 (73%) patients and on the opposite side in 7 (27%) (*P =* 0.007). From 33 patients with pure head tilt, columella deviation was observed in 19 patients, which was on the same side as the head tilt in 17 (89.5%) and in the opposite direction in 2 (10.5%) patients (*P *= 0.003). In patients with pure head tilt, one-sided nostril compression was found in 19 patients, which was on the opposite side of the head tilt in 17 (89.5%) and the same direction in 2 (10.5%) patients (*P *= 0.019). Among 33 patients with pure head tilt, cheek compression was seen in 21 patients, which was on the opposite side of the head tilt in 14 (66.6%) and the same direction in 7 (33.4%) patients (*P = *0.315). From 33 patients with pure head tilt, 12 (36.4%) patients exhibited all qualitative facial asymmetry manifestations (one-sided nostril compression, columella deviation, cheek and face compression).

### Group 2: Patients with pure head turn

The mean age of 58 patients with pure head turn was 16.0 ± 9.9 years (range, 2.5–38 years), which comprised of 27 (46.6%) males and 31 (53.4%) females. While 53 patients had DRS (42 esotropic [type I] and 11 exotropic [type II]), 5 patients had pure head turn due to unilateral upshoot in adduction [Figure 3]. DRS was found in the left eye of 47 (88.6%) patients, and in 6 (11.4%) patients it was observed in the right eye (*P* < 0.001).

Facial asymmetry was found in 50 (86.2%) patients with pure head turn, and 8 (13.8%) had a completely symmetric face (*P* < 0.001). In 58 patients with pure head turn, the mean amounts of the facial angle and relative facial size are shown in Table 1. The frequencies of “presence of facial asymmetry”, “one-sided nostril compression”, “columella deviation”, “one-sided cheek compression”, and “one-sided face compression” in patients with pure head turn are shown in Figure 4.

Fifty-eight patients had a pure head turn, forty-five of them had a relative facial size of > 1.01, face compression was on the opposite side of the head turn in 27 (60%) and on the same side in 18 (40%) patients (*P = *0.074). From 58 patients with pure head turn, columella deviation was observed in 43 patients, which was on the opposite side of the head turn in 32 (74.4%) and the same direction in 11 (25.6%) patients (*P = *0.002). In patients with pure head turn, one-sided nostril compression was found in 42 patients, which was in the same direction as the head turn in 27 (64.3%) and in the opposite direction in 15 (35.7%) patients (*P = *0.101). Among 58 patients with pure head turn, cheek compression was seen in 37 patients, which was on the opposite side of the head turn in 25 (67.6%) and the same direction in 12 (32.4%) patients (*P = *0.032). From 58 patients with pure head turn, 37 patients (63.8%) exhibited all qualitative facial asymmetry manifestations (one-sided nostril compression, columella deviation, cheek and face compression).

### Group 3: Control group 

The mean age of 64 orthotropic subjects, including 30 (46.9%) female and 34 (53.1%) male, was 17.7 ± 10.3 years (range 2.5–38 years). The patient and control groups were matched for age (*P *= 0.485). The means of facial angle and relative facial size in the control group are shown in Table 1. In the control group, the mean frequencies of “facial asymmetry”, “one-sided nostril compression”, “columella deviation”, “one-sided cheek compression”, and “one-sided face compression” are shown in Figure 4.

Of the 64 orthotropic subjects, 22 (34.3%) had facial asymmetry. In the control group, one-sided face compression was observed in 17 (26.6%) subjects with a relative facial size of > 1.01. From the 64 orthotropic subjects, one-sided nostril and columella deviations were seen in 18 (28.1%) and 8 (12.5%) cases, respectively. Only three (4.7%) orthotropic subjects had all qualitative facial asymmetry manifestations (one-sided nostril compression, columella deviation, cheek and face compression). There was no significant difference in one-sided face and nostril compression; and columella deviation between the right and left facial sides (*P *= 0.467, *P *= 0.166, and *P *= 0.116, respectively).

The mean facial angle was significantly higher in patients with pure head tilt as compared to pure head turn patients (*P *= 0.004, F = 0.927, t = 2.988, df = 90, Mean Difference = 0.589). Also, in patients with pure head tilt, the mean relative facial size was significantly higher than in pure head turn patients (*P *= 0.018, F = 1.140, t = 2.405, df = 90, Mean Difference = 0.008).

All qualitative facial asymmetry parameters (one-sided face, cheek, and nostril compression and columella deviation) and the frequency of facial asymmetry were significantly higher in head tilt and head turn patients as compared to orthotropic subjects (all *P* < 0.001, Chi-square test).

**Table 1:** The mean of relative facial size and facial angle in patients with pure head tilt, head turn, and orthotropic participants as control group.


**Parameters**	**Groups**	**Number**	**Minimum**	**Maximum**	**Mean ± SD**	**95% Confidence Interval for Mean**		P-value*
			**Lower bound**	**Upper bound**	
Facial angle (º)	Pure head tilt	33	0.40º	4.45º	1.78º ± 1.01º	1.42º	2.14º	< 0.001
	Pure head turn	58	0.00º	3.57º	1.19º ± 0.84º	0.97º	1.41º	
	Orthotropic subject	64	0.00º	2.85º	0.68º ± 0.77º	0.49º	0.87º	
Relative facial size	Pure head tilt	33	1.000	1.080	1.027 ± 0.018	1.021	1.034	< 0.001
	Pure head turn	58	1.000	1.072	1.018 ± 0.014	1.015	1.023	
	Orthotropic subject	64	1.000	1.050	1.009 ± 0.012	1.006	1.012	
	
	
*One way ANOVA								

##  DISCUSSION

The primary aim of this study was to compare the frequency of quantitative and qualitative facial asymmetry parameters in patients with head tilt versus head turn. The results have shown that the quantitative parameters were significantly higher in patients with pure head tilt as compared to those with pure head turn. As well, all facial asymmetry parameters and the frequency of facial asymmetry were significantly higher in the head tilt and head turn groups as compared to the control group.

Based on the results, the frequency of qualitative facial asymmetry parameters such as the one-sided face, cheek, and nostril compression and columella deviation was observed in more than 50% of patients with abnormal head posture. In line with the current study, another study performed by the same authors reported a higher amount of quantitative facial parameters (facial angle and relative facial size) and a higher frequency of qualitative parameters in DRS as compared with the control group.^[8]^ However, in that study, we evaluated facial asymmetry only in DRS. Other studies reported a high frequency of facial asymmetry in patients with multiple ocular causes of abnormal head posture.^[8,9,10,11,12,17,18]^ However, the main limitations of these studies include a lack of orthotropic subjects being used as a control group,^[9,10,17]^ using only qualitative methods,^[10,18]^ and only recording the presence of facial asymmetry.^[11,17,18]^ In addition to the mentioned limitations, all of them evaluated facial asymmetry according to the underlying disease of abnormal head posture, and they did not report characteristics of facial asymmetry in different types of abnormal head posture.^[8,9,10,11,12,17,18]^

Our findings show that the mean relative facial size and facial angle; and the frequency of facial asymmetry were higher in patients with pure head tilt in comparison with patients with pure head turn.^[7,10,19]^ Head tilt can change the balance of the facial sides, with one side being closer to the ground and the other side being farther away. However, this imbalance does not play an essential role in patients with pure turn because, in these patients, the head rotates horizontally.^[8]^

Although facial asymmetry often exists in conjunction with plagiocephaly from early childhood without head tilt, the effect of gravity would explain other main findings in patients with pure head tilt including columella deviation on the same side as the head tilt (toward the ground) and one-sided nostril compression on the opposite side (opening a nostril that is closest to the ground and compression of the other one). Due to head tilt, facial muscles stretch on the opposite side of the head tilt, and face compression occurs on the same side.^[7,10,16]^

The frequency of one-sided face and nostril compression did not have any significant difference between both facial sides in pure head turn patients. However, in most patients with pure head turn, columella was deviated on the opposite side of head turn. The main cause of this finding is unknown, however, some studies reported normal genetic pattern of development tends to divert their columella toward the midline.^[7,10]^

Knowing the frequency and type of facial asymmetry in patients with head tilt versus head turn seems important. Based on the results of this study, the head tilt group had a higher frequency of facial asymmetry than the head turn group. Therefore, examiners should be aware that the lack of appropriate treatment of abnormal head posture in patients with head tilt may be associated with the higher frequency of facial asymmetry as compared to patients with head turn. However, it is important to consider that ectopia may happen in patients with nonsyndromic craniosynostosis (e.g., plagiocephaly), non-paretic motility disorders because of an extorted position of the orbit and pulley.^[20,21]^

In some abnormal head posture causes such as superior oblique palsy, the abnormal head posture would manifest as head tilt or head turn or a combination of them. In a study by Turan et al, four different types of abnormal head posture in patients with superior oblique palsy was reported.^[4]^ Also, it is essential to know that according to Mitchell's study,^[22]^ 9% and Nucci's study,^[3]^ 13% of patients with abnormal head posture did not have any background disease or apparent reason for their condition. In this condition, regardless of the abnormal head posture causes, clinicians should be aware that the frequency of facial asymmetry in patients with head tilt may be more than the head turn group.

The clinicians can evaluate facial asymmetry in both quantitative and qualitative methods. We believe that the best way to find facial asymmetry is the quantitative measurement of facial asymmetry parameters. Even in one study by Goodman et al, only the amount of facial angle was considered as the amount of facial asymmetry in patients with ocular torticollis.^[9]^ In this study, facial angle > 3º was defined as facial asymmetry. Furthermore, we considered the amount of relative facial size > 1.01 as facial asymmetry, and it might be helpful for clinicians that this subtle difference could surprisingly be visible and easily detected.

The following points were considered for a proper comparison among the groups:

The age of patients and subjects among the three groups was matched.

All patients had congenital etiology of abnormal head posture.

For better control of genetic factors and environmental conditions on creating facial asymmetry, orthotropic subjects were selected from families of patients (siblings and cousins).

As the amount of head tilt cannot be compared with the amount of head turn, we calculated relative facial size and facial angle. Therefore, our findings can be compared appropriately among both patients and control groups.

Not measuring the degrees of head tilt and face turn from the normal positioning was the main limitation of this study. We also did not evaluate facial characteristics in patients with a combined head tilt and turn, which was another limitation of our study. As one of the main reasons for resolving abnormal head postures by strabismus surgery is the prevention of facial deformation,^[9]^ knowing about the frequency of facial asymmetry in various manifestations of abnormal head postures is essential. Therefore, a suggestion to other researchers is to conduct further studies in order to determine the prevalence of facial asymmetry in all types of abnormal head posture, such as combined head tilt and turn on the same side, combined head tilt and turn on the opposite side, chin elevation and depression.

We concluded that the quantitative parameters such as relative facial size and facial angle were significantly higher in patients with pure tilt as compared to those with pure head turn. Also, all facial asymmetry parameters and the frequency of facial asymmetry were significantly higher in the head tilt and head turn groups compared to the control group. The results of this study have shown that the abnormal head posture of pure head tilt was associated with a higher prevalence of facial asymmetry compared to that of pure head turn. Therefore, examiners should be aware that the lack of appropriate treatment in patients with head tilt may be associated with the higher frequency of facial asymmetry as compared to patients with head turn.

##  Declaration of Patient Consent

The authors certify that they have obtained all appropriate patients consent forms. In the form, the patients have given their consent for their images and other clinical information to be reported in the journal. The patients understand that their names and initials will not be published and due efforts will be made to conceal their identity, however, anonymity cannot be guaranteed.

##  Financial Support and Sponsorship

None.

##  Conflicts of Interest

None.
